# Necrotizing sialometaplasia as a cause of a non-ulcerated nodule in the hard palate: a case report

**DOI:** 10.1186/1752-1947-5-406

**Published:** 2011-08-23

**Authors:** Mônica Ghislaine Oliveira Alves, Dárcio Kitakawa, Yasmin Rodarte Carvalho, Luiz Antonio Guimarães Cabral, Janete Dias Almeida

**Affiliations:** 1 Department of Biosciences and Oral Diagnosis, São José dos Campos Dental School, Universidade Estadual Paulista - UNESP, São José dos Campos, São Paulo, Brazil

## Abstract

**Introduction:**

Necrotizing sialometaplasia is a benign, self-limiting and rare inflammatory disease which, on clinical and histological examination, mimics malignant neoplasms.

**Case report:**

We report the case of a healthy 25-year-old Caucasian woman with a three-week history of a painless lump on her hard palate. Oral examination revealed a nodule consisting of two lobules on the right side that measured 2.5 cm. Her mucosa was normal in color and a fluctuant area was detected in the posterior region upon palpation. Our patient was submitted to incisional biopsy and histopathological examination. The histological diagnosis was necrotizing sialometaplasia. The lesion had healed spontaneously after 30 days, with observed signs of involution of the nodule.

**Conclusion:**

Histopathological examination is necessary for the diagnosis of necrotizing sialometaplasia because the clinical features of this condition can mimic other diseases, particularly malignant neoplasms.

## Introduction

Necrotizing sialometaplasia is a benign, self-limiting and rare inflammatory disease of the minor salivary glands [1-6], which was first described as a distinct entity by Abrams *et al. *in 1973 [7]. Knowledge about the disease is required because it mimics malignant neoplasms on clinical and histological examination, particularly squamous cell carcinoma and mucoepidermoid carcinoma [2-4,6,8]. We report the clinical and histopathological features of a case of necrotizing sialometaplasia presenting initially without ulceration in a young adult woman.

## Case report

A healthy 25-year-old Caucasian woman was seen at our stomatology outpatient clinic with a three-week history of a lump on her hard palate, which was non-tender upon oral examination. Our patient reported the presence of a stabbing pain radiating to the region of the temporomandibular joint in the previous week. The patient was a dentist and made a self-diagnosis of an abscess.

**Figure 1:**
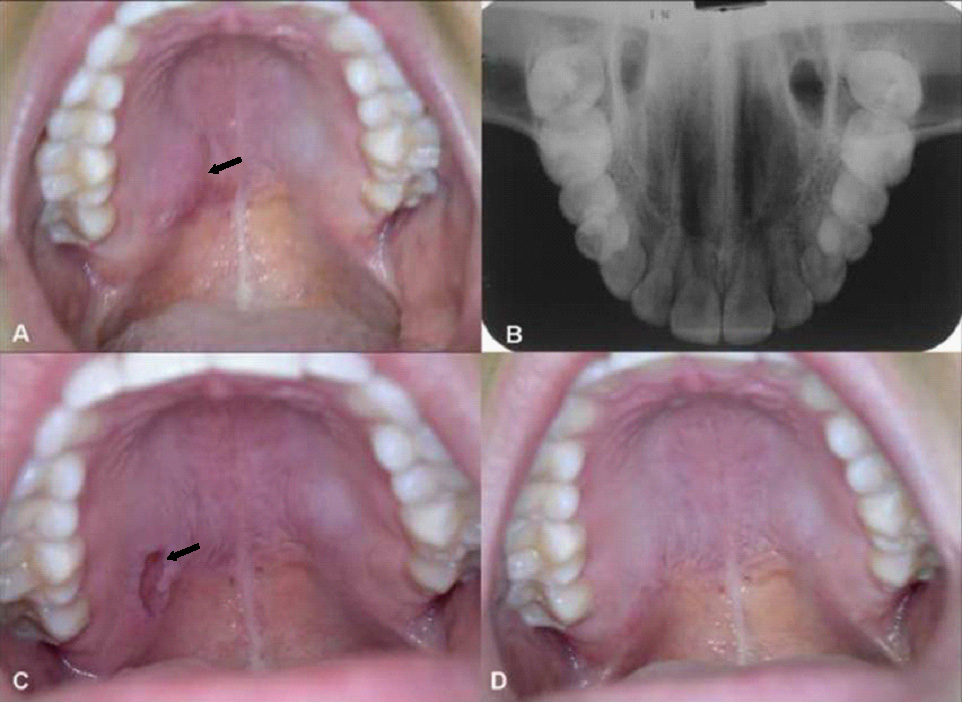
**Clinical features**. A: Submucosal nodule on the right side of the hard palate in the absence of mucosal alterations (continuous arrow). B: Occlusal radiograph showing no abnormalities. C: Ulceration in the biopsy area after 14 days (continuous arrow). D: Healed area after 30 days.

**Figure 2:**
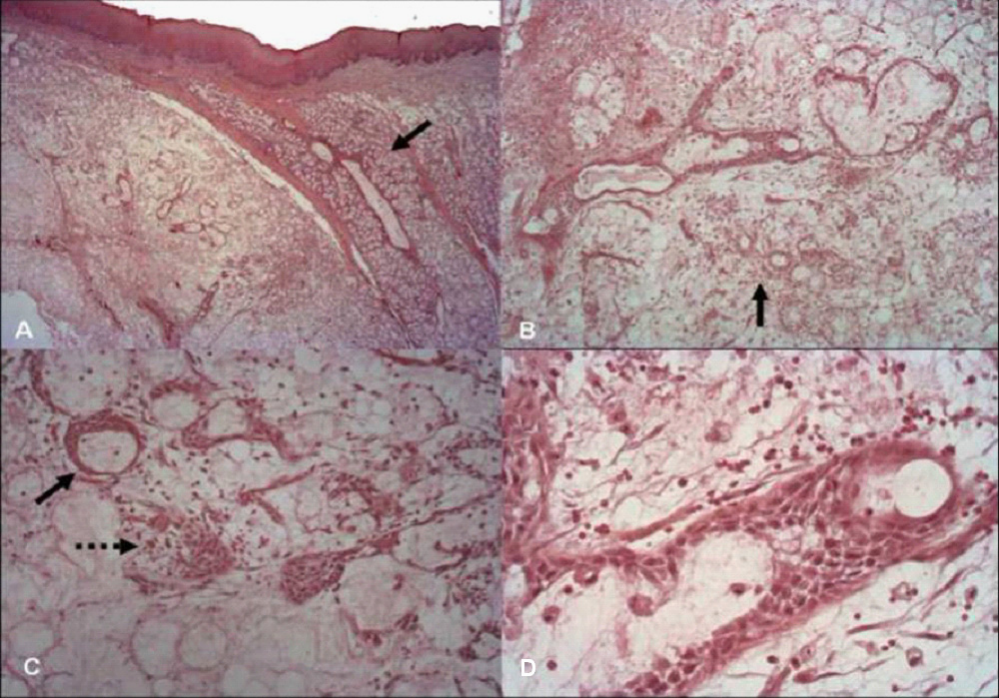
**Histopathological features (H&E staining)**. A: Preservation of the lobular architecture (25×) (continuous arrow). B: Atrophic broken acini with leakage of mucus and ductal dilatation (100×) (continuous arrow). C: Ducts showing squamous metaplasia (continuous arrow) and a moderate stromal mononuclear inflammatory infiltrate (dotted arrow) (200×). D: The same aspects as shown in B and C at 400× magnification.

Clinical examination revealed a submucosal nodule on the right side of her hard palate that measured almost 2.5 cm in its major diameter. The color of the mucosal surface was normal (Figure 1A) and a fluctuant area was detected in the posterior region upon palpation. Occlusal radiography revealed no abnormalities (Figure 1B). The first diagnostic hypothesis was malignant salivary gland tumor; most likely mucoepidermoid carcinoma considering the stabbing pain, duration of the lesion and palpation of a fluctuant area. An incisional biopsy was performed. Histological examination of the specimen revealed a mucosal fragment lined with parakeratinized stratified epithelium exhibiting mild hyperplasia. Several minor salivary gland lobules were found deep in the lamina propria, which were characterized by atrophic, sometimes broken acini, leakage of mucus, intraglandular ductal dilatation, and a moderate stromal mononuclear inflammatory infiltrate. Some lobules were necrotic, although the lobular architecture was preserved. The lobules were permeated by ducts with squamous metaplasia. Leakage of eosinophilic amorphous material was observed, which was intermingled with an intense mixed inflammatory infiltrate containing foamy macrophages. No signs of malignancy were found. The diagnosis was necrotizing sialometaplasia (Figure 2).

Seven days after surgery, the biopsy wound showed normal healing. Ulceration was noted in the biopsy area after 14 days (Figure 1C). The lesion had healed spontaneously after 30 days, with the observation of clinical signs of involution of the nodule (Figure 1D).

## Discussion

The exact etiology of necrotizing sialometaplasia is unknown, but ischemia of local blood supply in the salivary gland lobules is the most widely accepted theory. Causes of this ischemia include local trauma, local anesthesia, ill-fitting dentures, smoking, alcohol consumption, radiation, allergies, upper respiratory tract infection, intubation, surgical procedures involving the area [2,3,5,8], cocaine use [1], and chronic vomiting [5,9]. In the present case, the cause of the lesion could not be established since our patient did not report any of these conditions.

Necrotizing sialometaplasia can be found at any site that contains salivary glands [6], but mainly affects the minor salivary glands located in the hard palate [2,4,5,8]. The disease manifests as a deep-seated ulcer, measuring on average 1.8 cm in its major diameter [2]. Other less frequently involved sites include the maxillary sinus, retromolar pad, lower lip, tongue, oral mucosa, mucobuccal fold, tonsillar fossa, nasal cavity, incisive canal, larynx, and trachea. Involvement of the major salivary glands has been reported mainly after surgical interventions [2,3]. Bilateral involvement is rare [3]. Swelling is initially observed, followed by ulceration that may be accompanied by fever. Pain is a common symptom. Paresthesia in the affected area is rare [2-4].

Necrotizing sialometaplasia mainly affects white men, with a male-to-female ratio of two to one. The average age at diagnosis is 46 years [2,3], although the case of a two-year-old girl diagnosed with the disease has been reported in the literature [8]. In the present case, the disease was diagnosed in a woman whose age was below the range reported for the disease. Our patient was a dentist and had a history of palatal swelling that had appeared three weeks earlier and presented with stabbing pain in the absence of clinical alterations of the mucosa. These findings are important for the clinician, who must be aware that a swelling in the palate may not be an inflammatory process related to infection.

The typical manifestation of necrotizing sialometaplasia is a deep ulcer and the differential diagnosis includes granulomatous diseases such as syphilitic gumma and deep mycosis lesions, which may show a sharp demarcation. Opportunistic infections are common in patients with poorly controlled diabetes and may mimic necrotizing sialometaplasia [8]. In the present case, no ulceration was seen and the differential diagnosis was malignant salivary gland tumor, most likely mucoepidermoid carcinoma [2].

The microscopic findings of necrotizing sialometaplasia include coagulation necrosis of glandular acini, an inflammatory response, pseudoepitheliomatous hyperplasia of overlying epithelium, and maintenance of the lobular architecture [2-5,7,8]. Ductal squamous metaplasia and reactive fibrosis can be seen in older lesions [2-4,6]. Anneroth and Hansen [9] used histopathology to classify necrotizing sialometaplasia into five stages: infarction, sequestration, ulceration, reparative stage, and healed stage. During infarction, necrosis of the glandular acini predominates and culminates in the formation of the ulcer. At the beginning of the healing stage, proliferation of the overlying epithelium is observed, which is demonstrated microscopically by pseudoepitheliomatous hyperplasia. If infarction is limited, no sequestration occurs. Healing becomes evident by the phagocytic activity of histiocytes and neutrophils and the presence of granulation tissue [2,3]. In the present case, the biopsy was obtained at an early stage of the disease, a fact that may explain the absence of an ulcer.

Squamous metaplasia of the ductal epithelium, accompanied by pseudoepitheliomatous hyperplasia of the overlying epithelium, might be confused with squamous cell carcinoma [2] when viewed under the microscope, despite the presence of a minimum number of mitoses, pleomorphism, and hyperchromatism [3].

In the present case, the process was detected at the very early stage of the disease that is characterized by the absence of nodular ulcerated lesion. The ulcer that developed 14 days after biopsy showed spontaneous remission 30 days after its occurrence and involution of the nodule was observed.

Necrotizing sialometaplasia resolves spontaneously and the lesion heals by secondary intention within four to ten weeks. Therefore, no treatment is necessary [2,3,10]. Once the lesion has healed, recurrence or functional impairment is not observed [8]. A biopsy is necessary when the clinical findings indicate other diagnostic hypotheses [2], as observed in the present case.

## Conclusion

In conclusion, histopathological examination is necessary in cases of necrotizing sialometaplasia since the clinical features of this condition can mimic other diseases, particularly salivary gland tumors.

## Consent

Written informed consent was obtained from the patient for publication of this case report and any accompanying images. A copy of the written consent is available for review by the Editor-in-Chief of this journal.

## Authors' contributions

MGOA was a major contributor in writing the manuscript. YRC performed the histological examination. JDA, DK and LAGC analyzed and interpreted the patient data, performed the surgical procedures, and took the photographs. All authors read and approved the final manuscript.

## Competing interests

The authors declare that they have no competing interests.
